# Muscle-derived stem/progenitor cell dysfunction in *Zmpste24*-deficient progeroid mice limits muscle regeneration

**DOI:** 10.1186/scrt183

**Published:** 2013-03-25

**Authors:** Minjung Song, Mitra Lavasani, Seth D Thompson, Aiping Lu, Bahar Ahani, Johnny Huard

**Affiliations:** 1 Stem Cell Research Center, Department of Orthopaedic Surgery, University of Pittsburgh School of Medicine, 450 Technology Drive, Suite 206, Pittsburgh, PA 15219, USA; 2 Department of Bioengineering University of Pittsburgh Swanson School of Engineering, 300 Technology Drive, Suite 360B, Pittsburgh, PA 15219, USA; 3 Department of Microbiology and Molecular Genetics, University of Pittsburgh School of Medicine, 450 Technology Drive, Suite 523, Pittsburgh, PA 15219, USA

## Abstract

**Introduction:**

Loss of adult stem cell function during aging contributes to impaired tissue regeneration. Here, we tested the aging-related decline in regeneration potential of adult stem cells residing in the skeletal muscle.

**Methods:**

We isolated muscle-derived stem/progenitor cells (MDSPCs) from progeroid *Zmpste24*-deficient mice (*Zmpste24*^-/-^) with accelerated aging phenotypes to investigate whether mutation in lamin A has an adverse effect on muscle stem/progenitor cell function.

**Results:**

Our results indicate that MDSPCs isolated from *Zmpste24*^-/- ^mice show reduced proliferation and myogenic differentiation. In addition, *Zmpste24*^-/- ^MDSPCs showed impaired muscle regeneration, with a limited engraftment potential when transplanted into dystrophic muscle, compared with wild-type (WT) MDSPCs. Exposure of progeroid *Zmpste24*^-/- ^MDSPCs to WT MDSPCs rescued the myogenic differentiation defect *in vitro*.

**Conclusions:**

These results demonstrate that adult stem/progenitor cell dysfunction contributes to impairment of tissue regeneration and suggest that factors secreted by functional cells are indeed important for the therapeutic effect of adult stem cells.

## Introduction

Hutchinson-Gilford progeria syndrome (HGPS) is an autosomal dominant disease that involves premature aging, causing early death in childhood due to stroke or myocardial infarction. The patients have sclerotic skin, joint contractures, bone abnormalities, and growth impairment [1]. A point mutation of the lamin A gene (*LMNA*), which encodes lamin A protein, was found to be the main cause of HGPS [2,3]. Lamin A is a nuclear envelope protein that gives structural support to the nucleus and is involved in various cellular roles, such as gene expression and DNA replication [4,5].

Murine models of HGPS have been created by altering posttranslational modification steps of lamin A [6-8]. Young and colleagues [6] developed *Zmpste24 *knockout mice with many features common to HGPS [6]. ZMPSTE24 is a metalloproteinase required for cleaving the carboxylic group of prelamin A to create lamin A. By knocking out *Zmpste24*, prelamin A accumulates on the cell nuclear envelope, resulting in cellular blebbing [9,10]. The *Zmpste24*^-/- ^mice display accelerated aging, loss of weight, spontaneous bone fracture, cardiomyopathy, muscular dystrophy, muscle atrophy, and muscle weakness [6,7,9,11]. A recent study provides evidence that the skeletal muscles of *Zmpste24*^-/- ^mice exhibit impaired muscle contraction and neuromuscular performance [11].

Prelamin A has been shown to be involved in early steps of C2C12 myoblasts differentiation [12]. C2C12 myoblasts expressing mutated lamin A have also demonstrated a reduced capacity to undergo myogenic differentiation [13]. Downregulation of lamin A/C levels in myoblasts, by transfecting them with a mutant *Lmna *gene, isolated from *Lmna*-knockout mice, or from silencing RNA targeting A-type lamins, showed impaired differentiation kinetics and reduced differentiation potential [14,15]. These data provide evidence for a critical role of prelamin A in the early steps of muscle cell differentiation.

MDSPCs are an important population of adult stem cells isolated from skeletal muscle by using a modified preplate technique [16,17]. They show self-renewable potential and multilineage differentiation for myogenic, osteogenic, chondrogenic, and adipogenic lineages *in vitro *[16,18-21]. Prominent muscle regeneration has been observed with MDSPCs transplantation into a dystrophin knockout mouse model of Duchenne muscular dystrophy (*mdx*) [16,22,23]. Although adult stem cells play essential roles in maintaining tissue and organ function with self-renewal and multilineage differentiation potential, they also show age-dependent changes, such as decline in number and function similar to other somatic cells [24]. Given that stem cell exhaustion and loss of function with age may limit their muscle-regeneration potential, we investigated the impact of aging on MDSPC function by using progeroid *Zmpste24*-deficient mice, which exhibit accelerated aging and mimic HGPS.

## Methods

### *Zmpste24*^-/- ^mice

*Zmpste24*^-/- ^genotyping was performed with polymerase chain reaction (PCR) with oligonucleotides forward: 5'-TCACATGGAGTGAATGCTCTG-3' and reverse: 5'-AGTGAACACCAGGCCAGTTT-3' [6]. All animal experiments were performed in accordance with the Institutional Animal Care and Use Committee of the University of Pittsburgh.

### MDSPC isolation

MDSPCs were isolated from 8-week-old *Zmpste24*^-/- ^mice and WT littermates by using a modified preplate technique according to a previously established protocol [16,17]. MDSPCs were cultured in proliferation medium (PM) containing Dulbecco's modified Eagles medium (DMEM, high glucose) supplemented with 10% horse serum, 10% fetal bovine serum (FBS), 1% penicillin-streptomycin (all from Invitrogen, Grand Island, NY, USA), and 0.5% chick embryo extract (Accurate Chemical, Westbury, NY, USA), on collagen type I-coated flasks (Sigma-Aldrich, St. Louis, MO, USA). Cells were used between passages 20 and 30.

### Proliferation *in vitro*

The population doubling time (PDT) over a 72-hour period was measured from time-lapse images acquired through a live-cell imaging (LCI) system (Automated Cell Technologies, Inc., Pittsburgh, PA, USA), as previously described [25,26]. In brief, cells were plated at an initial density of 2,000 cells/well in collagen type I-coated 24-well plates, and images were acquired at 15-minute intervals over a period of 3 days by using an LCI system equipped with a 10× objective. Five images at each time point were randomly selected and analyzed, by using ImageJ software (v. 1.44p; National Institutes of Health, Bethesda, MD, USA), from three independent experiments on three distinct cell lines in each group.

### Myogenic differentiation *in vitro*

MDSPCs were seeded onto collagen type I-coated six-well plates (1 × 10^5 ^cells/well) and cultured for 2 days in PM. Myogenic differentiation was induced by replacing the PM with differentiation media (DMEM, 2% FBS, 1% penicillin-streptomycin) and cultured for 3 days. Immunostaining for fast myosin heavy chain (f-MyHC), a marker of terminal myogenic differentiation, was performed to stain differentiated muscle cells. Cells were fixed with -20°C methanol, blocked with 5% goat serum, and incubated at room temperature with primary mouse anti-MyHC fast (1:250; Sigma-Aldrich) and secondary biotinylated IgG (1:250; Vector Laboratories, Burlingame, CA, USA) each for 1 hour, and streptavidin-594 (1:500; Sigma-Aldrich) for 15 minutes. The nuclei were visualized by staining with DAPI (4', 6' diamidino-2-phenylindole, 100 ng/ml; Sigma-Aldrich) for 10 minutes. Fluorescent images were taken by using a Leica DMIRB inverted microscope equipped with a QImaging Retiga digital camera with Northern Eclipse software (v. 6.0; Empix Imaging, Inc., Cheektowaga, NY, USA). Total cell nuclei and nuclei within f-MyHC-positive myofibers, from at least 15 fields, each from three replica platings, were counted by using ImageJ. The myogenic differentiation was calculated as percentage of cells expressing f-MyHC (red) per total nuclei (DAPI, blue).

### Western blot

MDSPCs from WT and *Zmpste24*^-/- ^were lysed in Radio-Immunoprecipitation Assay (RIPA) buffer (Sigma-Aldrich). Protein concentration was measured with the Bradford protein assay reagent (Bio-Rad, Hercules, CA, USA). Equal amounts of protein from each sample were loaded on 10% SDS-polyacrylamide gels and run for 2 hours at 100 volts. Proteins were then transferred for 60 minutes by 100 volts to a nitrocellulose membrane (Millipore, Billerica, MA, USA) and blocked by 5% nonfat dry milk (Bio-rad) in Tris-buffered saline Tween-20 (TBST) for 1 hour. Samples were probed with goat N-terminal anti-lamin A/C (1:200, sc-6215), goat C-terminal anti-prelamin A (1:200, sc-6214), and rabbit anti-β-actin (1:200, all from Santa Cruz Biotechnology, Inc., Dallas, TX, USA), overnight at 4°C. After washing 3 times with TBST, the membrane was incubated with secondary antibody rabbit anti-goat IgG (H&L) coupled to horseradish peroxidase (HRP; Santa Cruz Biotechnology, Inc.) and mouse anti-rabbit IgG-HRP for 1 hour at room temperature. Blots were developed by using a SuperSignal West Femto with enhanced chemiluminescent substrate (1:100,000; Thermo Scientific, Rockford, IL, USA), and the bands were detected on x-ray film.

### Reverse transcriptase polymerase chain reaction (RT-PCR)

RNA was extracted from cultured cells by using TRIzol (Invitrogen) and a RNeasy Mini kit (Qiagen Inc., Hilden, Germany). The amount of RNA yield was quantified by using a Nano-Quant (Tecan, San Jose, CA, USA), and a total of 1 μg of RNA was reverse-transcribed into cDNA by using the Superscript III Reverse Transcriptase (Invitrogen), according to the manufacturer's instructions. Then, cDNA was mixed with GoTaq polymerase, dNTPs, and green GoTag reaction buffer (Promega, Madison, WI, USA) and amplified on a thermal cycler (Eppendorf Mastercycler proS), according to the manufacturer's instructions for 30 cycles at 58°C annealing temperature. PCR products were separated by using 2% agarose gels with 1% ethidium bromide. The gel images were captured with a BioRad Gel Doc system. The following primers were used: Stem cell antigen-1 (Sca-1), forward 5'-CCTACTGTGTGCAGAAAGAGC-3' and reverse 5'-CAGGAAGTCTTCACGTTGACC-3'; CD34, forward 5'-GCAGCTTTGAGATGACATCACC-3' and reverse 5'-CTCAGCCTCCTCCTTTTCACA-3'; myogenin, forward 5'-CTACAGGCCTTGCTCAGCTC-3' and 5'-TTGTGGGCGTCTGTAGG-3'; desmin, forward 5'-AACCTGATAGACGACCTGCAG-3' and reverse 5'-GCTTGGACATGTCCATCTCCA-3'. β-actin, forward 5'-GGGTCAGAAGGACTCCTATG-3' and reverse 5'-CTTTGATGTCACGCAGCACGATT-3' was used as a loading control.

### Coculture experiments

*Zmpste24*^-/- ^MDSPCs were plated in the lower compartment of Costar Transwell Permeable Supports (Corning, Tewksbury, MA, USA) in PM at a density of 3,000 cells/well in a 24-well collagen type I-coated plate. WT MDSPCs were seeded into 6.5-mm transwell membrane inserts at the same density in PM and placed above the *Zmpste24*^-/- ^MDSPCs. The 24-well plates containing the transwells were then placed in the LCI system for 72 hours to measure proliferation of the *Zmpste24*^-/- ^MDSPCs, as described earlier. As a control, each plate contained wells of *Zmpste24*^-/- ^MDSPCs with empty transwell membrane inserts. To measure the differentiation potential of *Zmpste24*^-/- ^MDSPCs after coculture, transwell inserts were removed after 72 hours, and the PM media was switched to differentiation media. After 2 to 3 days, myogenic differentiation of the cells was tested by immunostaining for f-MyHC, as described earlier. Changes in differentiation potential of *Zmpste24*^-/- ^MDSPCs was also tested by using conditioned media from the WT MDSPCs. WT MDSPCs were cultured for 2 days in 25 cm^2 ^collagen-coated flasks and were then treated with differentiation media for 3 days. The supernatant was collected and used as conditioned media for 8-week-old *Zmpste24*^-/- ^MDSPCs, whereas unconditioned differentiation media was used as a control.

### Cell transplantation

The 3 × 10^5 ^donor MDSPCs isolated from WT or *Zmpste24*^-/- ^mice were intramuscularly transplanted into the gastrocnemius muscles of 8- to 9-week-old *mdx*/SCID mice (C57BL/10ScSn DMD*mdx*/J/CB17-Prkdcscid/J; The Jackson Laboratory), an animal model of Duchenne muscular dystrophy that is also immunocompromised. Mice were killed 2 weeks after transplantation, and the gastrocnemius muscles (*n *= 3) were harvested, frozen in 2-methylbutane pre-cooled in liquid nitrogen, and cryosectioned (10 μm). Sections were immunostained for dystrophin, the cross-sectional area of dystrophin-positive myofibers was measured, and the distribution of the fiber areas was plotted as previously described [27,28].

### Statistical analysis

Statistical analyses were carried out by using Sigmastat (Jandel Scientific, v. 2.0, San Rafael, CA, USA) software package. The Student's *t *test or Mann-Whitney Rank Sum test was used for direct comparisons between groups. For multiple comparisons, the one-way ANOVA or the Kruskal-Wallis one-way ANOVA on ranks was applied. Pairwise multiple comparisons were performed by using the Tukey's Test after rank-based ANOVA. All values are expressed as the mean ± SD, and *P *< 0.05 was considered significant.

## Results

### MDSPCs from accelerated aged mice show defect in prelamin A processing and stem cell marker expression

**Figure 1:**
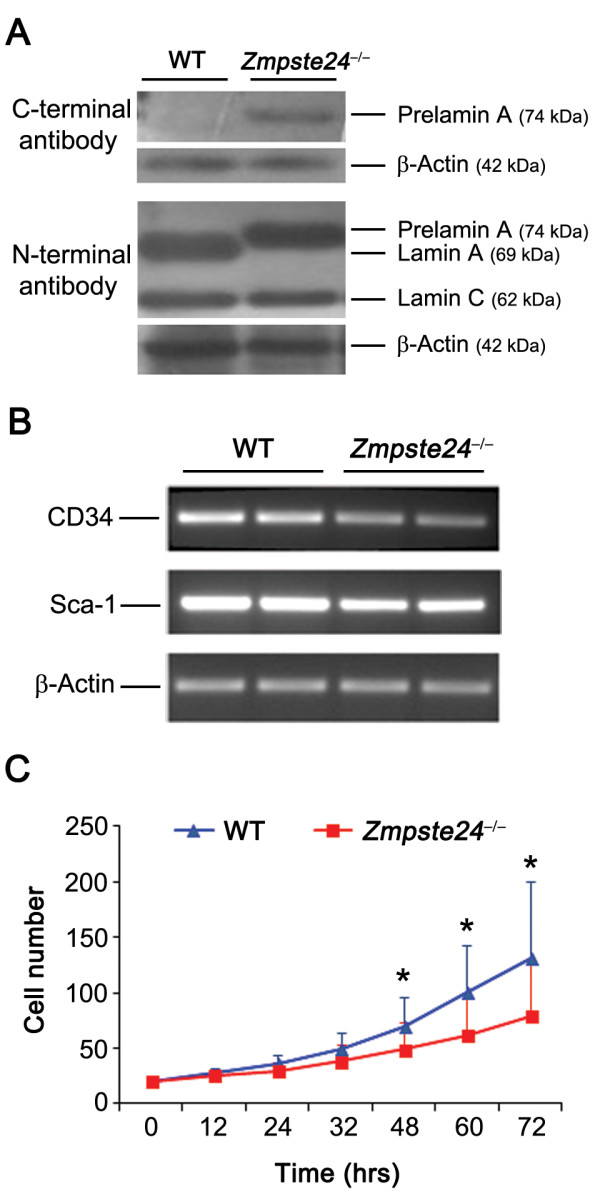
**Prelamin A processing, stem cell marker expression, and proliferation potential are affected in *Zmpste24*-deficient muscle-derived stem/progenitor cells (MDSPCs)**. **(A) **Immunoblot analysis to measure the expression of the C-terminal prelamin A and N-terminal lamin A/C in WT and *Zmpste24*^-/- ^MDSPCs relative to β-Actin. **(B) **RT-PCR to measure expression of stem-cell markers, CD34 and Sca-1, in MDSPC populations (*n *= three to four cell populations per group). **(C) **Proliferation of MDSPC populations was measured with a live cell-imaging system for 72 hours. Plotted are averages of normalized cell numbers at each time point calculated from the analysis of three cell populations per group, ± SD. *P < 0.001, Mann-Whitney Rank Sum test.

To determine whether a defect in lamin A/C processing, previously reported in *Zmpste24*-deficient mice [7,10], affects our stem/progenitor cells, MDSPCs isolated from 8-week-old WT and *Zmpste24*-deficient mice were evaluated for expression and distribution of lamin A/C and prelamin A (Figure 1A). An antibody specific to prelamin A and lamin A/C revealed accumulation of prelamin A and an absence of lamin A expression in the *Zmpste24*^-/- ^MDSPCs in comparison with WT MDSPCs. These results confirm the lack of prelamin A processing in *Zmpste24*-deficient MDSPCs, as previously reported in other cells isolated from these mutant mice [9,29]. Stem cell potential was also investigated by measuring the expression levels of CD34 and Sca-1 for each cell population [27]. *Zmpste24*-deficient MDSPCs show relatively lower expression levels of CD34 compared with WT MDSPCs; however, the expression levels of Sca-1 were similar in both groups (Figure 1B).

### *Zmpste24*^-/- ^MDSPCs show slower proliferation

Proliferation kinetics of MDSPCs isolated from WT and *Zmpste24*-deficient mice was measured *in vitro *by using an LCI system, and their numbers per image were manually counted. Analysis of images showed that the proliferation rate of WT and *Zmpste24*^-/- ^MDSPCs were similar for the first 24 hours (Figure 1C). However, *Zmpste24*^-/- ^MDSPCs started to show a trend toward a reduction in proliferation rate after 48 hours, which became significant at 72 hours compared with WT MDSPCs (*P < 0.05, Figure 1C). These data demonstrate that the proliferation capacity of MDSPCs diminishes in *Zmpste24*-deficient mice, as observed in aged MDSPCs [30].

### *Zmpste24*^-/- ^MDSPCs display limited myogenic differentiation capacity

**Figure 2:**
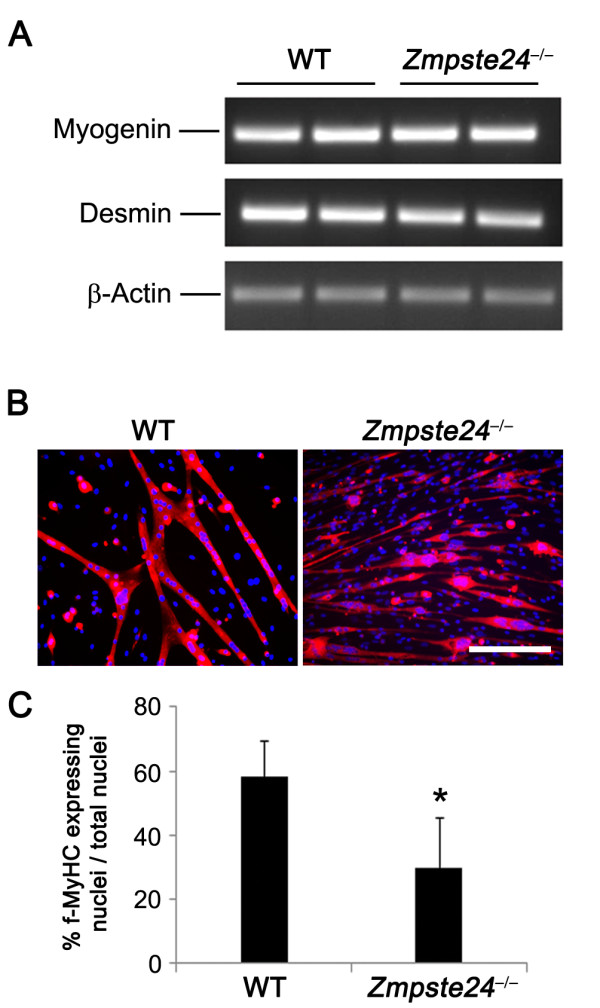
**Myogenic differentiation potential is reduced in *Zmpste24*^-/- ^muscle-derived stem/progenitor cells (MDSPCs)**. **(A) **RT-PCR to measure the expression of the myogenic differentiation markers, myogenin and desmin. **(B) **Representative images of *in vitro *MDSPC myogenic differentiation. MDSPC populations were grown to confluence and then switched to differentiation media. After 2 to 3 days, cells were immunostained for the terminal differentiation myogenic marker, f-MyHC (red). Scale bar, 100 μm. **(C) **Graph shows the myogenic differentiation (percentage of cells (DAPI, blue) expressing f-MyHC (red)) of MDSPCs isolated from *Zmpste24*^-/-^-deficient mice relative to young WT MDSPCs. Error bars indicate SD. *P < 0.001, Student's *t *test. Representative images are shown from three separate experiments using three independent MDSPC populations of each group.

To determine whether the myogenic differentiation capacity of *Zmpste24*^-/- ^MDSPCs is affected, the expression levels of essential proteins that play critical roles in regulating at least one component of myogenic terminal differentiation were investigated. Myogenin is a muscle-specific transcription factor involved in functional skeletal muscle development and is expressed mainly in intermediate stages of myogenesis [31]. Desmin is expressed at early stages of myogenic differentiation and continuously expressed near to terminal myogenesis [32]. Interestingly, the expression levels of myogenin and desmin were similar in *Zmpste24*^-/- ^MDSPCs compared with control WT MDSPCs, indicating that MDSPCs from aged mice have similar myogenic potential at the transcriptional level (Figure 2A). To test whether impairment may occur during the posttranslational step of myogenesis, cells were cultured to confluence and switched to differentiation media, and immunostained for the terminal myogenic differentiation marker, f-MyHC. The WT MDSPCs fused to form elongated multinucleated myotubes expressing f-MyHC, whereas *Zmpste24*^-/- ^MDSPCs formed significantly fewer and smaller myotubes, indicating impaired differentiation (*P < 0.001, Figure 2B, C). Collectively, these data establish that the function of the MDSPCs is compromised in *Zmpste24*^-/- ^mice, as observed in old WT MDSPCs [30].

### *Zmpste24*^-/- ^MDSPCs show limited muscle regeneration

**Figure 3:**
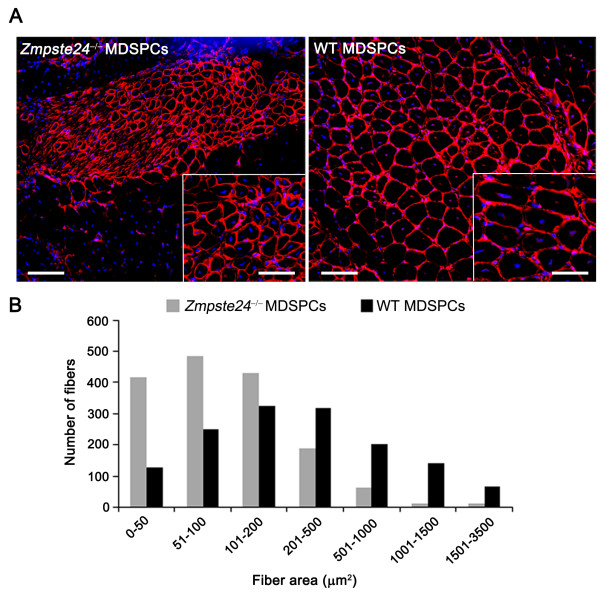
**Muscle regeneration of progeroid *Zmpste24*^-/- ^muscle-derived stem/progenitor cells (MDSPCs) is impaired *in vivo***. To measure MDSPC function *in vivo*, WT and *Zmpste24*^-/- ^MDSPCs were injected into the gastrocnemius muscle of 8-week-old *mdx*/SCID mice. **(A) **Representative images of sections of gastrocnemius muscle from dystrophic mice 14 days after injection immunostained for dystrophin (red) to identify regenerated myofibers. Scale bar, 100 μm; inset, 50 μm. **(B**) Quantification of the cross-sectional area of > 1,500 dystrophin-positive myofibers per group. The distribution of fiber size is indicated on the *x*-axis, representing increasingly more mature fibers with increased size (*P < 0.001; Mann-Whitney Rank Sum test at each size range except 100 to 200). Graph represents the results from injection of three to four *mdx*/SCID mice with three independent MDSPC populations per group.

To investigate the myogenic potential of progeroid *Zmpste24*^-/- ^MDSPCs *in vivo*, 3 × 10^5 ^viable MDSPCs isolated from WT and *Zmpste24*^-/- ^mice were injected into the gastrocnemius muscle of 8-week-old *mdx*/SCID mice, a mouse model of Duchenne muscular dystrophy that lacks dystrophin at the sarcolemma of muscle fibers [33] and is also immunoincompetent. The average number of regenerated dystrophin-positive myofibers did not significantly differ between the WT (625, *n *= 3) and *Zmpste24*^-/- ^(508, *n *= 3) MDSPCs (*P *= 0.80). However, a dramatic difference was noted in the average cross-sectional area of the regenerated myofibers in mice injected with *Zmpste24*-deficient MDSPCs compared with WT MDSPC-injected mice (Figure 3A). Most of the regenerated myofibers from the WT MDSPC transplantations had an area of > 500 μm^2^, indicative of significantly more mature regenerated myofibers. Conversely, dystrophin-positive myofibers in mice injected with *Zmpste24*^-/- ^MDSPCs predominantly had an area of ≤ 500 μm^2 ^(*P < 0.001, Figure 3B). Dystrophin-positive centronucleated myofibers of small size regenerated by *Zmpste24*^-/- ^MDSPC transplantation are perhaps due to fusion of donor cells with one another or with smaller host-regenerated myofibers. Collectively, the data in Figures 2 and 3 support the conclusion that the loss of stem cell function in the *Zmpste24*-deficient model of accelerated aging limits muscle regeneration.

### WT MDSPCs rescue the myogenic differentiation defect of *Zmpste24*^-/- ^MDSPCs

**Figure 4:**
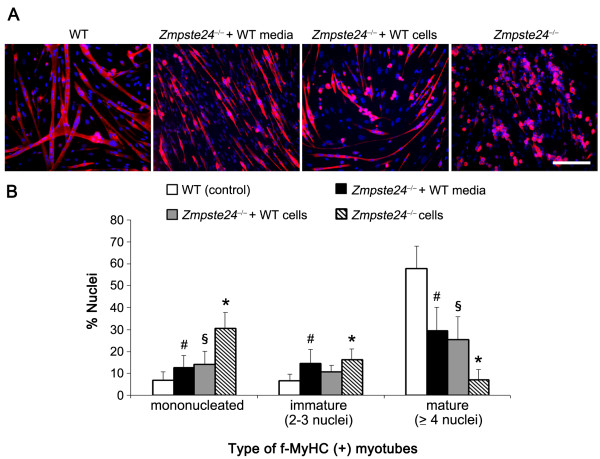
**Myogenic differentiation defect of *Zmpste24*^-/- ^muscle-derived stem/progenitor cells (MDSPCs) is rescued *in vitro***. **(A) **Representative images of myotube formation by *Zmpste24*^-/- ^MDSPCs, *Zmpste24*^-/- ^MDSPCs in the presence of culture media from WT MDSPCs, or cocultured with WT MDSPCs by using transwell inserts, f-MyHC (red); nuclear counterstain (DAPI; blue). Scale bar, 100 μm. **(B) **Plotted is the percentage of nuclei positive for f-MyHC in mononucleated (*P < 0.05; Tukey's test, relative to *Zmpste24*^-/- ^MDSPCs and WT MDSPCs), myotubes containing two to three nuclei (immature, ^#^*P *< 0.05; Tukey's test, in comparison with *Zmpste24*^-/- ^MDSPCs), and equal to or greater than four nuclei (mature, ^§^*P *< 0.05; Tukey's test, relative to *Zmpste24*^-/- ^MDSPCs and WT MDSPCs) in each group. Error bars, SD.

Next, we tested whether WT MDSPCs can restore the myogenic dysfunction of *Zmpste24*^-/- ^MDSPCs. MDSPCs isolated from *Zmpste24*-deficient mice were cocultured with the WT MDSPCs by using a transwell system, or in the presence of conditioned media from WT MDSPCs, and were switched to low-serum media to evaluate the myogenic differentiation. *Zmpste24*^-/- ^MDSPCs tend to fuse and form spheres (f-MyHC-positive) containing single or many nuclei (DAPI) (Figure 4A). In contrast, by using the conditioned media from WT MDSPCs or after coculturing the *Zmpste24*^-/- ^MDSPCs with WT MDSPCs, normal myotube formation was restored, indicated by numerous elongated multinucleated myotubes generated by the rescued *Zmpste24*^-/- ^MDSPCs (Figure 4A). The differentiation defect was reported by using the observation that *Zmpste24*^-/- ^MDSPCs formed more mononucleated and immature myotubes (myotubes containing two to three nuclei and expressing f-MyHC) compared with WT MDSPCs forming more mature myotubes (containing four or more nuclei; *P < 0.05). Analyzing the type of f-MyHC-expressing myotubes revealed a significant shift in the distribution to more mature myotube formation when *Zmpste24*^-/- ^MDSPCs were grown in the presence of the conditioned media isolated from WT MDSPC cultures (^#^*P *< 0.05, relative to WT MDSPCs) or cocultured with transwells containing the WT MDSPCs (^§^*P *< 0.05) relative to *Zmpste24*^-/- ^MDSPCs alone or WT MDSPCs (Figure 4B). Thus WT MDSPCs were able to rescue, at least in part, the myogenic differentiation capacity of MDSPCs isolated from *Zmpste24*^-/- ^progeroid mice.

## Discussion

Numerous studies have reported that mutation of lamin A, a major component of the nuclear envelope, has an adverse effect on skeletal muscle function and myoblast differentiation [7,9,11-14,34]; however, no studies examined earlier muscle stem/progenitor cells residing in muscle tissue. Because degenerative changes associated with progeroid syndromes may have a strong impact on stem cells, we hypothesized that the dysfunction of muscle stems cells in *Zmpste24*^-/- ^progeroid mice may contribute to the loss of the cells' ability to regenerate skeletal muscle. To evaluate this hypothesis, we isolated MDSPCs from *Zmpste24*^-/- ^mice showing nuclear lamina defects with accelerated aging phenotypes. Our results show that expression of stem cell markers decreased in MDSPCs isolated from skeletal muscle of *Zmpste24*^-/- ^progeroid mice in comparison with their WT littermates. Furthermore, MDSPC proliferation and myogenic differentiation was significantly impaired in *Zmpste24*^-/- ^MDSPCs relative to MDSPCs isolated from WT mice. This is consistent with our recent findings using naturally aged and excision repair cross-complementation group 1 (ERCC1)-deficient mice, a mouse model of progeroid syndrome, indicating that MDSPCs isolated from aged and progeroid mice showed loss-of-stemness properties including proliferation and multilineage differentiation [30]. A recent report also showed an alteration in the number and proliferation capacity of epidermal stem cells in *Zmpste24*^-/- ^progeroid mice [29]. Similar parallels were found in the vascular smooth muscles and mesenchymal stem cells (MSCs) compartment of induced pluripotent stem cells (iPSCs) from HGPS dermal fibroblasts [35]. Similarly, molecular and cellular identity, including the differentiation potential, were altered in human MSCs (hMSCs) isolated from HGPS patients [36].

Interestingly, the expression levels of myogenic markers, myogenin, and desmin, were not affected in MDSPCs isolated from *Zmpste24*^-/- ^mice, whereas the terminal myogenic differentiation marker, f-MyHC, was significantly impaired. This suggests that the dysfunction of MDSPCs is most likely due to changes in the posttranscriptional stages of myogenic differentiation. Recent studies suggested important findings regarding different signaling pathways involved in the regulation of stem-cell function. Signaling pathways essential for the maintenance of stem cell fate and function, such as Wnt in epidermal cells and microphthalmia transcription factor (Mitf) in melanocytes, has been shown to be defective in *Zmpste24*^-/- ^mice [29]. Lineage-specific differentiation of hMSCs was altered through the activation of the Notch signaling pathway, a pathway known to regulate stem-cell differentiation [36]. Therefore, future studies are necessary to elucidate the role of lamin A and possible changes in the signaling pathways of MDSPCs isolated from *Zmpste24*^-/- ^progeroid mice.

Our transplantation study showed that the muscle-regeneration potential of *Zmpste24*^-/- ^MDSPCs was significantly reduced in comparison to MDSPCs isolated from WT mice. This supports our hypothesis that loss of stem-cell function diminishes their regenerative potential. These results also agree with our recent findings that muscle from aged and progeroid ERCC1-deficient mice had significantly reduced regenerative capacity after cardiotoxin-induced injury [30]. This is consistent with the notion that defects in the adult stem-cell compartment, which accrue over time, contribute to the loss of tissue-regeneration capacity and homeostasis associated with aging. In addition to the cell-intrinsic defect, local microenvironment/niche and systemic environment have also been recognized to play a role in stem-cell aging [24]. Muscle stem cells interact with a microenvironment comprising of myofibers and the basal lamina. Therefore, the microenvironment of the *mdx*/SCID mouse skeletal muscle most likely could not support the effective regeneration of the progeroid MDSPCs, reinforcing the important role of a healthy microenvironment to promote successful tissue regeneration.

Notably, the ability of *Zmpste24*^-/- ^MDSPCs to undergo myogenic differentiation was rescued after coculture and in the presence of the WT MDSPC conditioned media. This result is similar to recent reports showing that WT MDSPCs rescue differentiation defects of MDSPCs isolated from naturally aged and progeroid ERCC1-deficient mice when cocultured [30]. These results demonstrate that dysfunction of progeroid stem cells can be rescued, at least in part, by exogenous factors secreted by the WT stem cells. Although the exact secreted factors involved have yet to be elucidated, several candidate proteins and signaling pathways are currently under investigation. Similar results were found *in vivo *by Conboy and colleagues [37] by using parabiosis, in which functional defects and the regenerative capacity of satellite cells in aged mice were restored by secreted factors from a young systemic environment. Together, these findings suggest the possibility of epigenetic changes of stem and progenitor cells in response to environmental cues. It will be of great interest to identify the factors secreted by tissue-specific host cells in response to transplanted donor cells.

Finally, limitation of progeroid MDSPCs to undergo myogenic differentiation, at least in part, may explain the muscle atrophy and weakness observed in *Zmpste24*^-/- ^progeroid mice. In addition, the reversal of the phenotypic defects of stem cells with coculture can provide future opportunities for therapeutic strategies.

## Conclusions

The present study demonstrates that MDSPCs isolated from *Zmpste24*^-/- ^progeroid mice are defective in their proliferation and differentiation capabilities in culture and during tissue regeneration. It remains unclear whether the defects in MDSPC function arise because of defects in the stem cells and/or the stem-cell niche. Therefore, future studies are needed to investigate the mechanism(s) that confer MDSPC dysfunction with aging. In addition, myogenic differentiation of defective MDSPCs was improved after coculturing the cells with young functional WT MDSPCs. Hence, identifying the factors secreted by the functional stem cells responsible for rejuvenation will be valuable for overcoming muscular degeneration commonly associated with muscular dystrophies and aging.

## Abbreviations

ANOVA: analysis of variance; cDNA: complementary deoxyribonucleic acid; DAPI: 4',6' diamidino-2-phenylindole; DMEM: Dulbecco's modified Eagle medium; dNTP: deoxyribonucleotide triphosphate; ERCC1: excision repair cross-complementation group 1; FBS: fetal bovine serum; f-MyHC: fast myosin heavy chain; HGPS: Hutchinson-Gilford progeria syndrome; hMSC: human mesenchymal stem cell; HRP: horseradish peroxidase; iPSC: induced pluripotent stem cell; LCI: live cell imaging; *LMNA*: lamin A gene; MDSPC: muscle-derived stem/progenitor cell; Mitf: microphthalmia transcription factor; PCR: polymerase chain reaction; PDT: population doubling time; PM: proliferation media; RIPA: radio-immunoprecipitation assay; RNA: ribonucleic acid; RT-PCR: reverse transcriptase polymerase chain reaction; Sca-1: stem cell antigen-1; SCID: severe combined immune deficiency; SDS: sodium dodecylsulfate; TBST: tris-buffered saline tween 20; WT: wild type.

## Competing interests

Johnny Huard receives consulting fees and royalties from Cook MyoSite Inc. All the other authors declare that they have no competing interests.

## Authors' contributions

MS designed experiments, carried out proliferation and myogenic differentiation assays, analyzed data, and drafted the manuscript. ML carried out coculture experiments and *in vivo *experiments, analyzed data, performed the statistical analyses, and wrote the manuscript. ST assisted with in *vivo *studies, image analysis, and revised the manuscript. AL isolated the stem-cell lines and provided assistance with experimental design. BA carried out all of the immunostaining, imaging, and quantification. JH supervised the design and execution of the experiments. All the authors read and approved the manuscript for publication.
